# A New, Atypical Case of Cobalamin F Disorder Diagnosed by Whole Exome Sequencing

**DOI:** 10.1159/000441134

**Published:** 2015-10-14

**Authors:** Panayiotis Constantinou, Mariella D'Alessandro, Paul Lochhead, Shalaka Samant, W. Michael Bisset, Catherine Hauptfleisch, John Dean, Deciphering Developmental Disorders Study Group

**Affiliations:** ^a^North of Scotland Regional Genetics Service, Ashgrove House, UK; ^b^Molecular Genetics Department, University of Aberdeen, UK; ^c^Department of Paediatric Gastroenterology, Royal Aberdeen Children's Hospital, Foresterhill, UK; ^d^Department of Neonatology, Aberdeen Maternity Hospital, Foresterhill, UK

**Keywords:** Cobalamin F disorder, *LMBRD1* mutation

## Abstract

Cobalamin F (cblF) disorder, caused by homozygous or compound heterozygous mutations in the *LMBRD1* gene, is a recognised cause of developmental delay, pancytopaenia and failure to thrive which may present in the neonatal period. A handful of cases have been reported in the medical literature. We report a new case, diagnosed at the age of 6 years through whole exome sequencing, with atypical features including prominent metopic suture, cleft palate, unilateral renal agenesis and liver abnormalities, which broaden the phenotypic spectrum.

## Established Facts

• Cobalamin F disorder is caused by homozygous or compound heterozygous mutation in *LMBRD1*.

• Typical presenting features include poor growth, developmental delay and macrocytic anaemia with neutropaenia and thrombocytopaenia.

## Novel Insights

• Cleft palate, renal agenesis, prominent metopic suture and neonatal liver dysfunction may be features of the disorder.

• Whole exome sequencing is a non-invasive route to diagnosis in rare developmental disorders where neonatal clinical and biochemical findings are inconclusive.

Disorders of intracellular cobalamin metabolism include methylmalonic acidaemia and homocystinuria. These inborn errors of metabolism can manifest with dysmorphism, developmental delay, encephalopathy and megaloblastic anaemia. Cobalamin F (cblF) is a co-factor in the vitamin B_12_ metabolism pathway, encoded by the *LMBRD1* gene on chromosome 6q13 [Rutsch et al., 2009]. Deficiency is inherited in an autosomal recessive fashion, characteristically associated with intrauterine growth restriction, failure to thrive, congenital heart disease, developmental delay, macrocytic anaemia, neutropaenia, thrombocytopaenia, hyperhomocysteinaemia and methylmalonic aciduria [Alfadhel et al., 2011]. It is usually diagnosed through urine organic acid and plasma amino acid analysis. Molecular analysis of *LMBRD1* can be used to confirm the genetic basis of the disease. We present a 6-year-old child with an atypical presentation of cblF disorder, diagnosed by whole exome sequencing (WES).

## Case Report

**Fig. 1:**
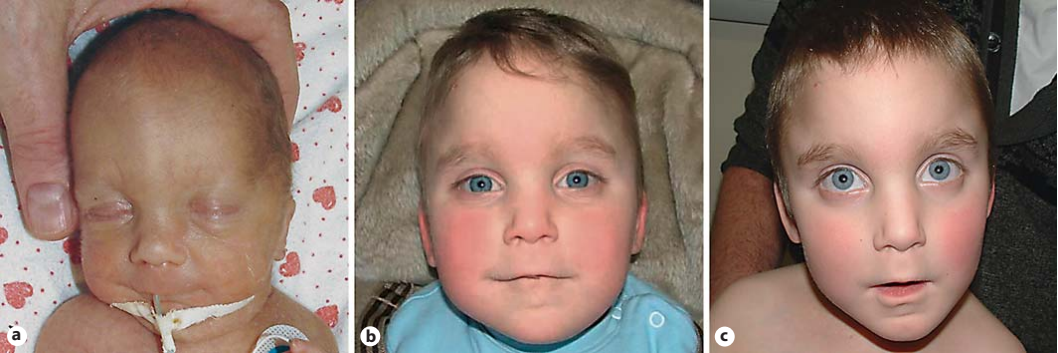
Frontal view of the facial characteristics. **a** Shortly after birth. **b** At age 3 years. **c** At age 6 years.

The proband was referred to our genetic service at the age of 1 month due to cleft palate, thrombocytopaenia and renal agenesis. The pregnancy history was notable for pregnancy-associated hypertension, for which the mother was managed with methyldopa. Prenatal ultrasonography demonstrated limb shortening and decreased liquor volumes. Delivery was by emergency lower segment Caesarian section at 32 weeks' gestation for fetal distress. APGAR scores were 8 at 1 min and 9 at 5 min after delivery. Growth parameters at birth were: weight 1,100 g (1.4th centile for gestational age), length 34 cm (<0.4th centile), and head circumference 28 cm (8th centile). Physical examination revealed a prominent metopic ridge, midline cleft soft palate, low-set ears, broad nasal tip, micrognathia and undescended testes with a right-sided inguinal hernia (fig. 1). Ophthalmological assessment revealed abnormal retinal streaking pigmentation. Imaging demonstrated thoraco-lumbar scoliosis and right renal agenesis. A small patent ductus arteriosus with mild peripheral pulmonary artery stenosis was identified, which resolved spontaneously by the age of 3 years. He had feeding difficulties and required parenteral nutrition during the first 6-8 weeks of life. Thrombocytopaenia and neutropaenia were evident, initially attributed to sepsis and treated with antibiotics. A bone marrow examination was normal apart from neutrophil maturation arrest at the metamyelocyte stage, which responded to granulocyte colony stimulating factor. He also had persistent conjugated hyperbilirubinaemia. A liver biopsy at 3 months of age revealed a paucity of bile ducts, but molecular analysis of *JAG1* for suspected Alagille syndrome was normal. Biochemical investigation at 6 weeks of age revealed a non-specific aminoaciduria with a slightly increased plasma homocysteine, in addition to peaks of methylmalonic acid, p-hydroxyphenyllactic acid and p-hydroxyphenylpyruvic acid on organic acid analysis. These findings were attributed to hepatic insult and inadequate vitamin supplementation while on parenteral nutrition. A single dose of intramuscular hydroxocobalamin was given and folate supplementation was instituted by tube feeding, continuing until the age of 3 years. Repeat biochemical studies at 2 and 3 months of age demonstrated an improvement in the biochemical profile (table 1). At this time, care was transferred to another team and permanent enteral feeding was commenced by percutaneous gastrostomy. As the initial blood abnormalities appeared to have resolved, further diagnostic investigation was not pursued at this stage.

Severe global developmental delay was apparent on follow-up. Unaided sitting was first recorded at around 3 years of age, and walking, with a wide-based gait, was first recorded at around 6 years of age. A mild intention tremor was noted from the age of 4 years. There was no speech on the last review at age 10, but he also had bilateral sensorineural deafness and communicated using some simple Makaton signing. He had proportional short stature with a height of 116 cm (<0.4th centile), weight 23.8 kg (5th centile), and a head circumference of 49 cm (<0.4th centile).

**Fig. 2:**
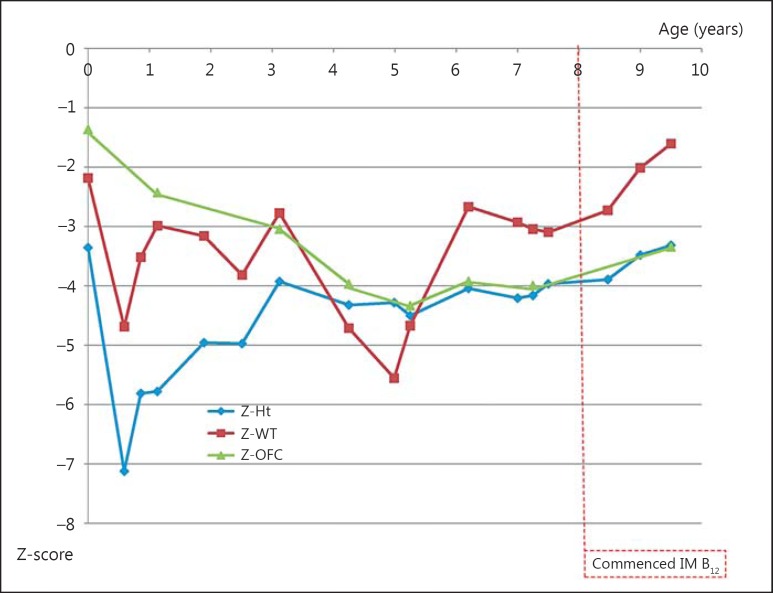
Growth parameters in the proband before and after institution of intramuscular (IM) hydroxocobalamin.

Array comparative genomic hybridisation analysis (Nimblegen CGX array v1.0) was normal as was a standard karyotype. At 6 years of age, he was entered into the Deciphering Developmental Disorders Study and WES was performed [methods described in the Deciphering Developmental Disorders Study, 2015]. This demonstrated a homozygous pathogenic mutation c.1056delG; p.Leu352fs*18 in the *LMBRD1* gene, which was subsequently confirmed by Sanger sequencing. Heterozygosity for this mutation was confirmed in both parents. No other potentially pathogenic variants were detected in any known development gene, no regional homozygosity was detected in the exome data using BCFtools v1.0, and no evidence of isodisomy was seen. After establishing the molecular diagnosis, biochemical studies were performed, revealing modestly elevated plasma homocystine and urine methylmalonic acid levels. Regular intramuscular hydroxocobalamin supplementation was instituted at this time, resulting in normalisation of the biochemical findings (table 1). There has been no change in his developmental status in the 2 years since starting therapy although growth parameters have improved slightly (fig. 2).

## Discussion

We present a new case of cblF disorder, presenting in an atypical manner with a prominent metopic suture, cleft palate, unilateral renal agenesis, feeding difficulties, and early liver abnormalities, which was diagnosed late by the use of WES. To date, a total of 15 cases have been reported in the literature [Rosenblatt et al., 1986; Shih et al., 1989; MacDonald et al., 1992; Wong et al., 1992; Waggoner et al., 1998; Rutsch et al., 2009; Gailus et al., 2010; Miousse et al., 2011; Oladipo et al., 2011] in addition to a recent case of combined cblF/cblG disorder suggestive of digenic inheritance [Farwell Gonzalez et al., 2015]. The typical clinical phenotype of cblF disorder is failure to thrive and feeding difficulties, developmental delay and haematological features including megaloblastic anaemia, neutropaenia and thrombocytopaenia (table 2). All but one of the previously described cases carry the c.1056delG; p.Leu352fs*18. Of the 7 cases that are homozygous for this mutation, the majority displayed developmental delay, haematological findings and failure to thrive, though their phenotypes were otherwise variable. Hepatic involvement and prominent metopic suture or dysmorphism as found in our patient have been infrequently described [Oladipo et al., 2011, Rutsch et al., 2009]. Cleft palate has not been described previously in cblF deficiency, but has been reported with other disorders of vitamin B_12_ and folic acid metabolism [Natsume et al., 1998; Weingärtner et al., 2007; Boyles et al., 2008]. Renal tubulointerstitial dysfunction has been described in disorders of cobalamin metabolism [Morath et al., 2013] as has reduced renal growth [Kruszka et al., 2013]. Renal agenesis has, however, not been described.

Early institution of intramuscular hydroxocobalamin corrects the biochemical abnormalities of hyperhomocysteinaemia and methylmalonic acidaemia and appears to limit the severity of cognitive and neurological impairment [Alfadhel et al., 2011]. Although late institution of intramuscular hydroxocobalamin corrected the biochemical profile in our patient, there has unfortunately been no effect on the severity of the patient's developmental delay, though there appears to be a trend of improvement in growth paramaters. The initial biochemical abnormalities might have guided towards the diagnosis, but were instead attributed to the effects of parenteral nutrition as they were partly corrected with modification of the regimen. In retrospect, the resolution of the haematological abnormalities also coincided with the institution of enteral nutrition and vitamin supplementation, but did not recur when vitamin supplementation was subsequently reduced. By virtue of his continued gastrostomy feeding, some vitamin supplementation has continued throughout his childhood, and this may explain why the usual haematological and biochemical features have not re-appeared. It should be noted that both prematurity and parenteral nutrition are associated with hepatic biochemical abnormalities [Schutzman et al., 2008]. We cannot be certain how much the cblF disorder contributed to the hepatic dysfunction in this case. This study supports the utility of a genomic testing approach to make an early diagnosis for neonates with a complex clinical presentation, as has been previously suggested elsewhere [Saunders et al., 2012].

The first 1,133 family trios from the Deciphering Developmental Disorders Study have now been reported, with an overall diagnostic yield of 27% [Deciphering Developmental Disorders Study, 2014; Wright et al., 2015]. One of the outcomes from the initial analysis has been the broadening of the phenotypic spectrum of previously well-described monogenic developmental disorders. The importance of high-quality or deep clinical phenotyping of rare diseases in the genomic era cannot be underestimated. It will improve our understanding of the natural history of these disorders, and may reveal findings that will affect clinical management. As in this case, it may reveal new genotype-phenotype correlations, giving a better understanding of the molecular bases of developmental disorders and hopefully facilitating the development of targeted therapies.

In summary, this new case of cblF deficiency, diagnosed via WES, broadens the phenotypic spectrum of the condition. It is likely that the incorporation of WES into clinical practice will highlight wider phenotypic variability in previously defined disorders.

## Statement of Ethics

The study has UK Research Ethics Committee approval (10/H0305/83, granted by the Cambridge South REC, and GEN/284/12 granted by the Republic of Ireland REC).

## Disclosure Statement

The authors have no conflicts of interest to declare.

## Figures and Tables

**Table 1:** Biochemical findings in the proband

Age	Plasma						Urine		Comments
	MMA (<10 μmol/mmol creatinine)	cysteine (20–80 μM)	methionine (10–60 μM)	homocystine (0–1 μM)	homocysteine (0–20 μM)	B_12_ (200–700 ng/l)	MMA (<10 μmol/mmol creatinine)	homocystine (<1 μmol/mmol creatinine)	
6 w				**29**			**530**		modestly elevated plasma phenylalanine and tyrosine

2 mo				**11**	**170**	>**1,000**	**304**	**96**	after supplementation
3 mo				**4**	**107**		**55**	**31**	

7.75 ys			28	<1	**33**	202	**30**	**5**	at molecular diagnosis after instituting hydroxocobalamin
8.1 ys	4	39	29	**2**	11	>**2,000**		**16**	
8.5 ys	**12**	40	34	<1	13			**13**	
8.75 ys	5	34	34	1	13			**12**	
9 ys	<1	45	20	<1	14		<1	**2**	
9.5 ys		40	19	<1	17				
9.8 ys	<1	42	29	1	14			**12**	

MMA = Methylmalonic acidaemia; mo = months; w = weeks; ys = years. Values in bold print represent abnormal results..

**Table 2:** Clinical features of cblF disorder described in the literature compared to features seen in our patient

Features	Number of affected patients	Our patient
Developmental delay	7	+
Failure to thrive	7	+
Haematological features	6	+
Congenital cardiac anomalies	6	+
Small for gestational age	6	+
Stomatitis +/– glossitis	5	
Gastric upset	3	
Dental anomalies	3	
Feeding difficulties	3	+
Microcephaly	2	+
Seizures	2	
Hypotonia	2	
Torticollis	1	
Pes equinovarus	1	
Tracheoesophageal fistula	1	
Intrauterine growth retardation	1	+
Arthritis	1	
Hepatic involvement	1	+
Rash	1	
Recurrent infection	1	
Facial dysmorphism	1	+
Recurrent apnoea	1	
Encephalopathy	1	
Cleft palate	0	+
Unilateral renal agenesis	0	+
